# Phenotypic Recovery of a *Heterobasidion* Isolate Infected by a Debilitation-Associated Virus Is Related to Altered Host Gene Expression and Reduced Virus Titer

**DOI:** 10.3389/fmicb.2021.661554

**Published:** 2021-10-14

**Authors:** Muhammad Kashif, Jaana Jurvansuu, Rafiqul Hyder, Eeva J. Vainio, Jarkko Hantula

**Affiliations:** ^1^Natural Resources Institute Finland, Helsinki, Finland; ^2^Department of Biology, University of Oulu, Oulu, Finland

**Keywords:** mycovirus, coinfection, virus transmission, growth rate, conifer pathogen

## Abstract

The fungal genus *Heterobasidion* includes forest pathogenic species hosting a diverse group of partitiviruses. They include the host debilitating Heterobasidion partitivirus 13 strain an1 (HetPV13-an1), which was originally observed in a slowly growing *H. annosum* strain 94233. In this study, a relatively fast-growing sector strain 94233-RC3 was isolated from a highly debilitated mycelial culture of 94233, and its gene expression and virus transcript quantities as well as the genomic sequence of HetPV13-an1 were examined. The sequence of HetPV13-an1 genome in 94233-RC3 was identical to that in the original 94233, and thus not the reason for the partial phenotypic recovery. According to RNA-seq analysis, the HetPV13-an1 infected 94233-RC3 transcribed eight genes differently from the partitivirus-free 94233-32D. Three of these genes were downregulated and five upregulated. The number of differentially expressed genes was considerably lower and the changes in their expression were small compared to those of the highly debilitated original strain 94233 with the exception of the most highly upregulated ones, and therefore viral effects on the host transcriptome correlated with the degree of the virus-caused debilitation. The amounts of RdRp and CP transcripts of HetPV13-an1 were considerably lower in 94233-RC3 and also in 94233 strain infected by a closely related mildly debilitating virus HetPV13-an2, suggesting that the virus titer would have a role in determining the effect of HetPV13 viruses on their hosts.

## Introduction

Fungal viruses (mycoviruses) are found in a broad range of fungal taxa including Ascomycota and Basidiomycota as well as early diverging fungal lineages, such as Chytridiomycota, Blastocladiomycota, Neocallimastigomycota, Zoopagomycota, and Mucoromycota (Ghabrial and Suzuki, 2009; Pearson et al., 2009; Ghabrial et al., 2015; Sutela et al., 2019, 2020; Myers et al., 2020). Viruses are transmitted among fungal strains by cell to cell contacts (hyphal anastomosis) and sexual or asexual spores (Ghabrial and Suzuki, 2009; Vainio et al., 2015b; Kashif et al., 2019). Mycoviruses usually cause asymptomatic infections. Hollings (1962) reported for the first time, debilitated growth of *Agaricus bisporus* mycelium caused by mycovirus infection on artificial agar media. Recently mycoviruses have also been reported to cause phenotypic alterations and hypovirulence (reduced virulence) in their hosts (Huang and Ghabrial, 1996; Preisig et al., 2000; Márquez et al., 2007; Yu et al., 2010; Hyder et al., 2013; Xiao et al., 2014; Vainio et al., 2018b). The hypovirulence responses have been well studied in *Cryphonectria parasitica* infected by Cryphonectria hypovirus 1 (Allen and Nuss, 2004; Deng et al., 2007; Dawe and Nuss, 2013; Eusebio-Cope et al., 2015). Genetically different fungal strains may also react differently to the same mycovirus strain (Hyder et al., 2013; Kim et al., 2015; Vainio et al., 2018b).

*H. annosum* s.lat. Species complex is considered as one of the most destructive groups of fungal pathogens of conifer forests in the Northern Hemisphere where these fungi cause root and butt rot diseases (Garbelotto and Gonthier, 2013). There are two geographical groups in this complex including three European species – *H. annosum*, *H. parviporum,* and *H. abietinum* – causing infections preferably on pine, spruce (*Picea abies* and others), and fir species, respectively (Niemelä and Korhonen, 1998; Dai and Korhonen, 1999), and two North American species – *H. irrugulare* and *H. occidentale* – infecting predominantly pines or overlapping host ranges of fir, spruce, and hemlock, respectively (Otrosina and Garbelotto, 2010; Garbelotto and Gonthier, 2013). For all these species, the primary infection is initiated by fungal spores landing and germinating on newly cut conifer stumps, or stem and root wounds, and continues as secondary infection *via* root contacts to neighboring trees (Stenlid and Redfern, 1998). They are also capable of both necrotic and saprotrophic growth. *H. irregulare* was the first *Heterobasidion* species to be characterized for complete genome sequence (Olson et al., 2012; Garbelotto and Gonthier, 2013), and thereafter also genomes of *H. annosum* (Sillo et al., 2015; Choi et al., 2017), *H. occidentale* (Liu et al., 2018), and *H. parviporum* (Zeng et al., 2018) have been characterized. There are different preventive control methods practiced against these root rot fungi, but truly curative methods are urgently needed. For this purpose, the use of mycoviruses has been suggested as a highly potential biocontrol option (Vainio and Hantula, 2016; Vainio et al., 2018b).

About 15% of *Heterobasidion* strains host dsRNA mycoviruses (Ihrmark, 2001; Vainio et al., 2015a; Vainio and Hantula, 2018) but the frequency of virus infections is considerably higher at forest sites with numerous *Heterobasidion* disease centers and thus ubiquitous mycelial contacts (Vainio et al., 2015b; Hyder et al., 2018). Up to 70% of Heterobasidion dsRNA virus infections are caused by Heterobasidion RNA virus 6 (HetRV6) (Vainio et al., 2012) belonging to the virus family *Curvulaviridae*, but also viruses belonging to genera *Alphapartitivirus, Betapartitivirus,* and family *Mitoviridae* have been observed (Vainio and Hantula, 2016; Vainio et al., 2018a,b), and actually partitiviruses form a major share of the viral species diversity in *Heterobasidion* spp. (Vainio et al., 2011, 2012; Kashif et al., 2015; Vainio et al., 2015a). The dsRNA genomes of partitiviruses are composed of two essential independently encapsidated segments coding for an RNA-dependent RNA polymerase (RdRp) and a coat protein (CP) (Nibert et al., 2014: Vainio et al., 2018a).

Although most *Heterobasidion* partitiviruses are cryptic, there are also strains causing variable and debilitating phenotypes on at least some of their hosts (Hyder et al., 2013; Jurvansuu et al., 2014). The best described of them is Heterobasidion partitivirus 13 strain an1 (HetPV13-an1) causing up to 90% reduction of its host’s growth rate (Vainio et al., 2018b; Kashif et al., 2019). There is also a closely related virus, HetPV13-an2 (Kashif et al., 2015; Hyder et al., 2018), which does not seem to cause noticeable effects on its natural host *H. annosum* S45-8 (Hyder et al., 2018).

The mechanisms behind the debilitating effects by HetPV13-an1 are not clear, although transcriptome analysis revealed considerable changes in *Heterobasidion* gene expression (Vainio et al., 2018b). This study is based on our observation of a fast-growing sector isolate of the original host strain of HetPV13-an1, *H. annosum* 94233, that seemed to have recovered spontaneously although the virus was still present in its hyphae. We aimed to investigate how the host can recover from the virus-induced growth retardation and the relevance of this phenomenon to the potential use of the virus as a biocontrol agent by (1) sequencing of the virus genome to determine the possibility of viral mutations, (2) analyzing the sector isolate’s gene expression to find changes that had occurred in the host, (3) quantifying viral genomes and transcripts in the recovered and debilitated isolates to determine whether changes had occurred in the virus quantity, and (4) analyzing the ratio of RdRp and CP transcripts of HetPV13-an1 among the isolates to test whether excess amount of viral polymerase transcripts correlates with a debilitated host phenotype as suggested earlier (Jurvansuu et al., 2014). A conspecific but apparently cryptic strain HetPV13-an2 was included in the phenotypical testing and analysis of viral transcript quantities to assess the effects of natural minor sequence variations to symptom severity.

## Materials and Methods

### Fungi and Viruses

**Figure 1:**
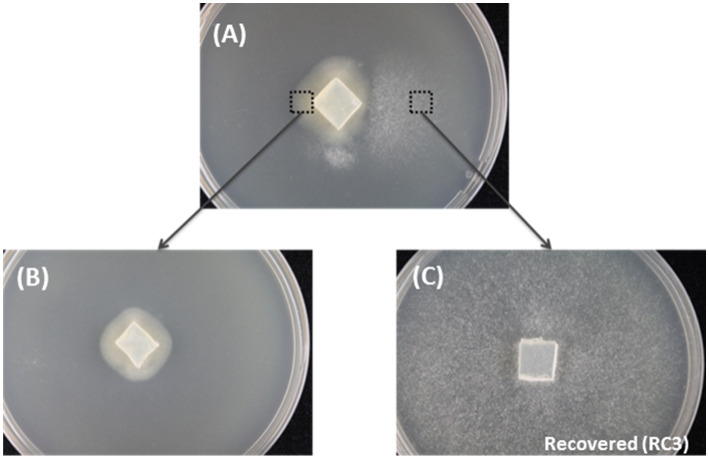
The spontaneous recovery of HetPV13-an1 infected fungal isolate as shown by mycelial morphology. **(A)** The original mycelium with slow and fast growth on the left and right side of the dish, respectively. **(B)** Mycelia transferred from the left side of the original mycelium. **(C)** Recovered 94233-RC3 transferred from the right side of the original mycelium.

HetPV13-an1 (GenBank accession: KF963177-78) is naturally hosted by *H. annosum* 94233 (Kashif et al., 2015; Vainio et al., 2015a). 94233-RC3 is a fast-growing sector isolate from 94233 (Figure 1) which was detected and stored in 2013 and then refreshened and restored in 2017 and 2018 at +4°C. The originally stored strain of 94233-RC3 as well as the restored strain in 2017 had lost the virus during the storage by 2021. Fortunately, the recently stored sample 2018 had retained the virus and could be used for further analysis. However, also one of the replicates regrown from this vial was found to have lost HetPV13-an1 and was named as 94233-RC3-0. In addition, two other isolates designated as 94233-RC1 and 94233-RC2 appeared to have recovered but returned quickly to their original slow-growing phenotype and were not analyzed further. The recovered HetPV13-an1 infected sector isolate 94233-RC3 was studied for its growth rate and gene expression in 2013. The strain was then stored at 4°C and moved back to +20°C only recently, after which it was found to have been cured of HetPV13-an1 in two of the three vials available: Vials from 2013 to 2017 had lost the virus by 2021, whereas a vial from 2018 had retained the virus and was selected for further analysis. However, also one of the replicates regrown from this vial was found to have lost HetPV13-an1 and was named as 94233-RC3-0.

*H. annosum* S45-8 is naturally coinfected by HetPV13-an2 (KF963179-80) (Kashif et al., 2015) and HetPV7-an1-b (KY859975-76) (Hyder et al., 2018). S45-8-PV13-an2 is a derivative of S45-8 cured of HetPV7 by single hyphal tip isolation. 94233-32D and S45-8-0 are previously and newly created partitivirus-free derivatives of 94233 (Vainio et al., 2018b) and S45-8, respectively, from which partitiviruses have been removed by a thermal treatment. Strain 94233-PV13-an2 was created by a transmission of HetPV13-an2 to 94233-32D by hyphal anastomosis on artificial medium.

### mRNA Sample Preparation and RNA-Sequencing

We analyzed the effects of virus infection on the gene expression of 94233-RC3 as compared to an isogenic partitivirus-free strain *H. annosum* 94233-32D. The fungal strains were grown on cellophane covered modified orange serum (MOS) plates (Müller et al., 1994) for 1week. Mycelia were collected and homogenized in TRI-Reagent (Molecular Research Center Inc., United States) using the Fast-Prep FP120 homogenizer (JT Baker, Holland) with quartz sand grains as recommended by the manufactures. The sample was precipitated with isopropanol and total RNA was further purified with E.Z.N.A Fungal RNA Kit (Omega Bio-tek), and RNA was resuspended into DEPC-treated water (G. Biosciences, United States). RNA quantity and quality were checked with NanoVue (GE healthcare, United States) and bioanalyzer 2,100 (Agilent technologies, United States). Each analysis was performed with three biological replicates. For RNA-seq, the TruSeq RNA Library Prep kit v2 (Illumina Inc.) was used to prepare the samples. The process involved poly(A) selection with oligo(dT) beads without the depletion of rRNA. The sequencing was conducted with an Illumina HiSeq2500 instrument using paired-end sequencing chemistry with 50-bp read length (Bioinformatics Core of Turku Centre for Biotechnology, University of Turku and Åbo Akademi University).

### dsRNA Extraction, RT-PCR, and Genome Sequence Determination

The dsRNA was extracted from 14days old fungal mycelia cultivated on cellophane membrane-covered MOS agar plates. Isolation of dsRNA was conducted according to Jurvansuu et al. (2014). Briefly, mycelia were homogenized and thereafter, RNA was isolated by phenol–chloroform (1:1) and chloroform–isoamylalcohol (24:1) extractions followed by precipitation by adding ethanol and NaCl at final concentrations of 15% and 100mm, respectively, and then adding cellulose fibers (medium) (Catalog no. C6288, Sigma-Aldrich, St. Louis, MO, United States). The precipitant was then moved to SigmaPrepTM spin columns (Sigma-Aldrich, United States) and washed. After eluting, the dsRNA was precipitated with ethanol. The dsRNA genome segments were excised from the gel and purified with RNAid Kit (MP Biomedicals, United States) and used in RT reaction.

T4RNA adapters were ligated and RT reactions set up using the RevertAid H minus M-MuLV reverse transcriptase (Thermo Scientific). The single primer amplification technique (Lambden et al., 1992) was used with previously described modifications (Tuomivirta and Hantula, 2003; Vainio et al., 2011; Kashif et al., 2015) to determine dsRNA sequences from isolate 94233-RC3. RT-PCR amplification was conducted using DyNAzyme DNA polymerase (Thermo Scientific) and specific primers (Kashif et al., 2015) as described before (Vainio et al., 2011). PCR products were sequenced at Macrogen Inc., South Korea^1^ using an Applied Biosystems 96-capillary ABI 3730xl DNA analyzer.

### Bioinformatics

The gene expression analysis based on RNA-seq (Illumina sequencing) was performed by the Bioinformatics Core of Turku Centre for Biotechnology, University of Turku and Åbo Akademi University as described in Vainio et al. (2018b). The resulting Illumina sequence reads were aligned against the reference genome of *H. irregulare*, named at the time as *H. annosum* v.2.0 at the Joint Genome Institute (JGI).^2^ The read alignment was against the *H. annosum* v.2.0 reference genome using TopHat version 2.0.10 (Kim et al., 2013). Sequence annotations were based on known genes from *H. annosum* v.2.0 reference genome using HTSeq tool v.0.5.4p3 (Vainio et al., 2018b).

The data were normalized to make the values comparable across the sample set and the counts were normalized by the TMM normalization method of the edgeR R/Bioconductor package. Summary of the mapping statistics is provided in Supplementary Table S1. The expression level of each gene was analyzed by the number of sequenced reads mapped to the sequence of the reference gene, and differentially expressed genes (DEGs) were determined according to criteria used in Vainio et al. (2018b) based on their fold changes (FC) over a value of 4 and modified *t*-test value of p (=false-discovery-rate value of p) of 0.001 (*p*<0.001).

### Validation of Gene Expression Data Using RT-qPCR

In order to simultaneously validate the results of RNA-Seq and to make comparison to the results in previous study (Vainio et al., 2018b) on the gene expression differences caused by HetPV13-an1 to strain 94233, the same 28 DEGs encoding various cellular functions as in Vainio et al. (2018b) were subjected for the RT-qPCR analysis of 94233-RC3 (Supplementary Table S2). Three independent biological replicates of each fungal sample (*H. annosum* 94233/32D and 94233-RC3) were incubated for 1week at 20°C and total RNA was extracted as described above for mRNA sequencing. The complementary DNA (cDNA) was diluted to a half-sample volume of water prior their use in RT-qPCR. The primers have been described in Vainio et al. (2018b), and three internal reference genes were used to normalize the results and primers as described by Raffaello and Asiegbu (2013) for RNA polymerase III transcription factor, Alfa tubulin, and Actin along with the 28 target genes.

RT-qPCR was carried out using EvaGreen^®^ qPCR Mix Plus (Solis BioDyne, Estonia), 2μl of cDNA and 1μl (10μm) of each primer in a total volume of 20μl in the 36-well rotor of Rotor-GeneQ (Qiagen, United States) by following the manufacturer’s instructions. Two replicates for each biological sample were prepared for each RT-qPCR reaction. Cycling conditions were as follows: pre-incubation at 95°C for 15min, denaturation at 95°C for 15s, using specific annealing temperatures for primers (Vainio et al., 2018b) for 20s, and extension at 72°C for 20s. Additionally, melting curve profile was analyzed to test the quality and specificity of the reactions. Relative gene expression (fold change) as expression ratios of samples to controls (normalized with three reference genes) were analyzed with relative expression software tool Rest2009^3^(Pfaffl et al., 2002) based on comparative quantitation analysis data. Relative expression (RT-qPCR) corresponds to log_2_ fold change of expression ratios measure by take-off values normalized with three reference genes.

### Quantification of Viral Transcripts and Genome Segments

#### Total RNA Isolation and cDNA Synthesis

Total RNA was isolated from fungal mycelia after 1week growth on MOS agar plates as described in Jurvansuu et al. (2014). In short, fungal mycelia were collected and total RNA was extracted by TRI-Reagent. The RNA pellet was eluted into DEPC-treated water and the concentration and purity of the isolated RNA were analyzed by NanoVue (GE healthcare, United States). cDNA was made from 2μg of DNase I treated total RNA using RevertAid First Strand cDNA Synthesis Kit (Thermo Scientific, United States) and random hexamer primers (Thermo Scientific, United States).

#### RT-qPCR Quantification for Virus Transcripts

Diluted cDNAs to half volume were used before using them in quantitative-PCR (qPCR). EvaGreen^®^ dye (Solis BioDyne, Estonia) was used in qPCR on Rotor-GeneQ (Qiagen, United States) as recommended by the manufacturer. PCR primers were used as follows: GAPDH as a reference gene and specific primers based on RdRp and CP genomic segments (proteins) of two virus strains including HetPV13-an1 and HetPV13-an2 (Vainio et al., 2015a) were used for virus transcripts. The purified plasmids of cloned genes of CP and RdRp of these viral strains (Jurvansuu et al., 2014; Kashif et al., 2019) were then used in absolute quantification as shown by Jurvansuu et al. (2014). The normalization of the viral transcript levels was done using the host GAPDH as a reference gene (Raffaello and Asiegbu, 2013) and the absolute quantities of RNA transcripts or virus copy number were calculated using a standard curve.

#### Phenotype Testing by Growth Rate Experiments

The effects of single viral infections by HetPV13-an1 or HetPV13-an2 were assessed by measuring the growth rate difference between pairs of isogenic HetPV13 infected and uninfected strains on 2% MEA plates at 20°C as described by Kashif et al. (2019). The growth rate effects of HetPV13-an1 were measured using derivatives of host strain 94233 and those of HetPV13-an2 using derivatives of host strains 94233 and S45-8. Each inoculum was a circular plug of 0.5cm diameter, which was picked from a fresh mycelial culture (1–2week old depending on the apparent growth rate), grown on 2% MEA plate and placed at the center of a 2% MEA plate. Twelve independent biological replicates were prepared for each isolate and fungal growth was measured every second day after mycelial growth was initiated 3days post-inoculation and continued until the mycelium covered the plate. The fungal growth was measured with a digital planimeter (Planix 10S, Tamaya) and statistical analysis was done using *t*-test in Microsoft Excel 2010 (Supplementary Table S3).

## Results

### Recovered Phenotype of Strain 94233

Three fast-growing sectors of *H. annosum* strain 94233 hosting HetPV13-an1 were initially observed in year 2013 and isolated as depicted in Figure 1. Two of the isolates returned quickly to their original slow-growing phenotype, but the third one, 94233-RC3, seemed to be stable in room temperature during several months. The presence of HetPV13-an1 in this fast-growing isolate was verified by RT-qPCR with specific primers 13an1RdRpF and 13an1RdRpRev as described earlier (Vainio et al., 2015a). The three mitoviruses previously shown to be hosted by *H. annosum* 94233 (Vainio et al., 2018b; Vainio, 2019) were also retained in 94233-RC3 as revealed by mapping of the RNA-Seq reads.

### Sequencing of the Virus Genome to Determine the Possibility of Virulence Reducing Mutations

**Figure 2:**
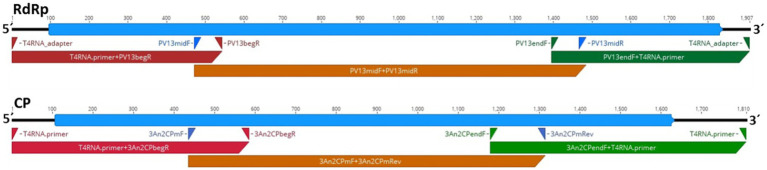
Schematic presentation of the genome organization of HetPV13-an1 (94233-RC3) showing the predicted coding regions for the RNA-dependent RNA polymerase (RdRp) and CP (blue) and the location of primers and corresponding amplification products (red, brown, and green) used for genome sequence determination.

The genome sequence of HetPV13-an1 in the *H. annosum* 94233 sector isolate 94233-RC3 (accession numbers MW115956 and MW115957) was 100% identical with the previously determined sequence for HetPV13-an1 (KF963177-78). Therefore, the observed phenotypical change was not caused by mutations in the virus genome sequence. Figure 2 shows a schematic presentation of the genome characterization scheme used. Each sequence site was covered by direct Sanger sequencing reactions including three PCR reactions each for CP and RdRp in two replicates, respectively.

### Analysis of Host Gene Expression

The transcriptome analysis of the sector isolate 94233-RC3 was conducted simultaneously with that of the partitivirus-free strain *H. annosum* 94233/32D and the debilitated original isolate 94233 included in our earlier study (Vainio et al., 2018b). Here, we first compared the expression of the sector isolate to the primary results on the transcriptome of 94233-32D (cured of HetPV13-an1) and thereafter compared the degree of observed differences to the corresponding changes in 94233 (with HetPV13-an1). After those analyses, the sector isolate was stored at 4°C.

The RNA-seq analysis of 94233-RC3 involved three biological replicates each of which produced ~16–22 million reads (Supplementary Table S1). The raw sequencing reads are deposited in the NCBI GenBank SRA data archive under the submission number SAMN16435702 (BioProject PRJNA362289). The annotation of identified transcripts was made based on the reference genome *Heterobasidium annosum* (v2.0) and resulted in identifying putative functions for eight genes containing five upregulated and three downregulated transcripts based on the same criteria as in Vainio et al., 2018b on the expression ratio (fold change) between the compared sample groups and FDR-adjusted value of p for the comparison between the sample groups (Table 1; Supplementary Table S4).

**Table 1:** Summary of RNA-seq analysis of *H. annosum* 94233-RC3 and 94233 infected with HetPV13-an1 and 94233/32D, which is a partitivirus-free isogenic strain.

	FC^d^	PType^e^	*p*-value	MeanExprs^f^	Tot^g^	Up^h^	Down^i^
13an1^a^ vs. ctr	4.000	FDR	0.001	0.125	683	276	414
RC3^b^ vs. ctr^c^	4.000	FDR	0.001	0.125	8	5	3

Three biological replicates were used for each strain.

a HetPV13-an1.

b 94233-RC3.

c 94233-32D.

d Expression ratio (fold change) between the compared sample groups, which is the lowest selection criteria or threshold limit of 4 and 2.5 to compare two groups (94233 and RC3-94233).

e FDR-adjusted value of p for the comparison between the sample groups.

f mean RPKM expression value.

g Total number of differentially expressed genes.

h Upregulated genes.

i Downregulated genes.

**Figure 3:**
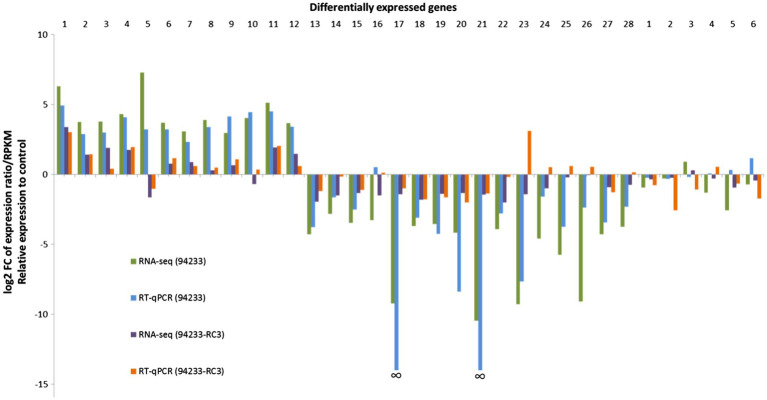
94233-RC3-transcriptome RNA-seq data and qPCR validation. The columns relate to fold changes in gene expression of HetPV13-an1 hosting *H. annosum* strains 94233 and 94233-RC3, each was compared to the HetPV13-an1-free control 94233-32D. Names and gene id numbers of the 28 and then 6 RNAi-related target genes are listed (Supplementary Table S2). The relative expression of RNA-Seq corresponds to log_2_ fold change of reads per kb of transcript per million mapped reads (RPKM). The data for HetRV3-an1 (94233) are taken from Vainio et al. (2018b).

The transcriptome of the sector isolate 94233-RC3 differed much less from that of the 94233-32D than the original virus-hosting strain 94233. Based on the number of reads related to each gene, only a total of eight transcripts were affected by HetPV13-an1 in 94233-RC3 (683 affected in 94233), of which five (276) were up- and three (414) downregulated (Table 1). All of the five genes upregulated in 94233-RC3 were upregulated also in 94233. In the case of the three downregulated genes, however, two of the genes were found to be almost five times less affected in 94233-RC3 than in 94233. Overall, the level of up- and downregulation of the host transcripts due to the HetPV13-an1 infection were considerably lower in 94233-RC3 than in 94233 (Figure 3).

The validation of 94233-RC3 gene expression by RT-qPCR mostly agreed with the RNA-seq data but there was also a significant exception: target gene (TG) 23 with clear down- and upregulation in RNA-seq and RT-qPCR, respectively (Figure 3). In addition, TGs 10, 24, 25, 26, and 28 for 94233-RC3 were found to be slightly downregulated based on RNA-seq but in RT-qPCR validation only negligible upregulation was observed (Figure 3; Supplementary Table S2). Target genes 17 and 21 did not amplify at all from *H. annosum* 94233 and therefore the fold change was not shown by RT-qPCR in Figure 3 (Vainio et al., 2018b).

### Qualitative Comparison of Gene Expressions of 94233-RC3 and 94233-32D in Relation to Biopathways

Probable gene functions affected by HetPV13-an1 in the sector isolate 94233-RC3 were assessed by comparing its transcript levels to the cured strain 94233-32D by RNA-Seq (Supplementary Table S4). The most prominent differences in the gene expression between HetPV13-an1 hosting sector isolate 94233-RC3 and the partitivirus-free strain 94233-32D were the three DEGs estExt_Genewise1Plus.C_080637, Hetan1.EuGene10000523, and e_gw1.12.503.1 that showed significant upregulation with FCs of 588, 387, and 180, respectively. These genes were related to putative Myosin class II heavy chain, Farnesyl cysteine-carboxyl methyltransferase (P450), and Sorbitol dehydrogenase proteins, corresponding to functions related to (1) fungal hyphal growth, septation, and conidial germination, (2) monooxygenase activity, and (3) secondary metabolites biosynthesis, transport, and catabolism, respectively. The functions of the two other upregulated genes are not known.

The difference in downregulated genes in the HetPV13-an1 infected 94233-RC3 was much smaller than in upregulated genes. DEG genesh1_kg.03__767__4245_1_CCOZ_CCPA_CCPB_CCPC_EXTA was downregulated 10-fold, but its function is not known. However, DEGs Hetan1.e_gw1.5.283.1 and Hetan1.estExt_fgenesh2_pg.C_100320 were downregulated less than 5-fold and code for putative L-fuculose-phosphate aldolase (fucA) and phenol 2-monooxygenase, respectively. The first of these functions is related to fructose and mannose metabolism, whereas the second one affects detoxification and aromatic compound metabolism.

RNAi-related genes are of a special interest because their functions are related to defense against mycoviruses. In 94233-RC3, none of these genes were up- or downregulated based on the RNA-Seq according to the criteria used here (and in Vainio et al., 2018b). However, in RT-qPCR analysis, two of the RNAi-related genes had significantly lower FC-values of −5.95 and−2.12, respectively. They code for a dicer (e_gw1.03.2068.1) and an argonaute gene (e_gw1.02.2254.1) (Supplementary Table S2). Thus, the effect of HetPV13-an1 on the viral RNAi-based defense of 94233-RC3 was quite low.

### The Most Differentially Expressed Genes in 94233-RC3 Compared to 94233

The gene expression was in general significantly less affected by HetPV13-an1 in 94233-RC3 than in the original 94233 (Vainio et al., 2018b). However, the most differentially expressed genes in the sector isolate, estExt_Genewise1Plus.C_080637, Hetan1.EuGene10000523, and e_gw1.12.503.1, had FCs 588, 387 and 180 in 94233-RC3 and 871, 202 and 225 in 94233, respectively, suggesting that HetPV13-an1 was still able to modify considerably the most seriously affected genes in the sector isolate.

### Analysis of the Ratio of HetPV13-an1 RdRp and CP Among the Isolates

**Figure 4:**
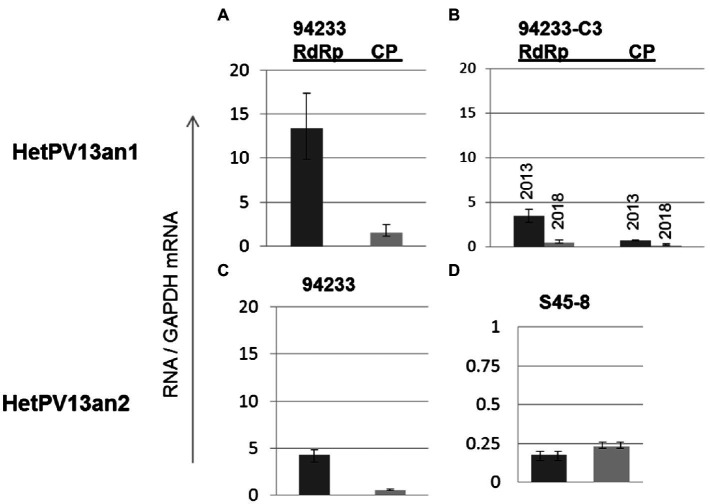
The variation in viral CP and RdRp transcript levels in *H. annosum* strains infected by partitiviruses. **(A)** Strain 94233 with its natural viral infection HetPV13-an1, **(B)** fast-growing sector isolate 94233-RC3 with infection by HetPV13-an1 before (2013) and after storage (2018), and **(C,D)** the amounts of viral transcripts of HetPV13-an2 in 94233/32D and in its native host (S45-8). Note: The scale for mRNA transcript levels is different in D due to significantly lower levels of expression.

The quantification of viral CP and RdRp mRNA transcripts using RT-qPCR revealed that the amounts of HetPV13-an1-encoded transcripts before storing 94233-RC3 were considerably lower in 94233-RC3 than in 94233 (Figures 4A,B) and differed between the two genes. The amounts of RdRp were 3.5 and 13.4 times of that of GAPDH RNA in 94233-RC3 and 94233, and the corresponding values for CP were 0.45 and 1.5, respectively. However, the RdRp and CP amounts reduced to 0.71 and 0.14 times of that of GAPDH RNA after long storage (2013–2021) of 94233-RC3. Moreover, the quantities of transcripts for RdRp and CP in 94233-RC3 were only 26 and 32% in 2013, respectively, of that in 94233, which further reduced in 94233-RC3 to 5 and 9% after long storage, respectively (Figures 4A,B). Similarly, the transcript levels of HetPV13-an2 RdRp and CP were 32 and 36% of that observed for HetPV13-an1 in 94233. However, HetPV13-an2 in its native host S45-8 produced significantly lower titer of the virus-an2 and showed more equal amounts of both transcripts from its genome segments (Figures 4C,D).

**Figure 5:**
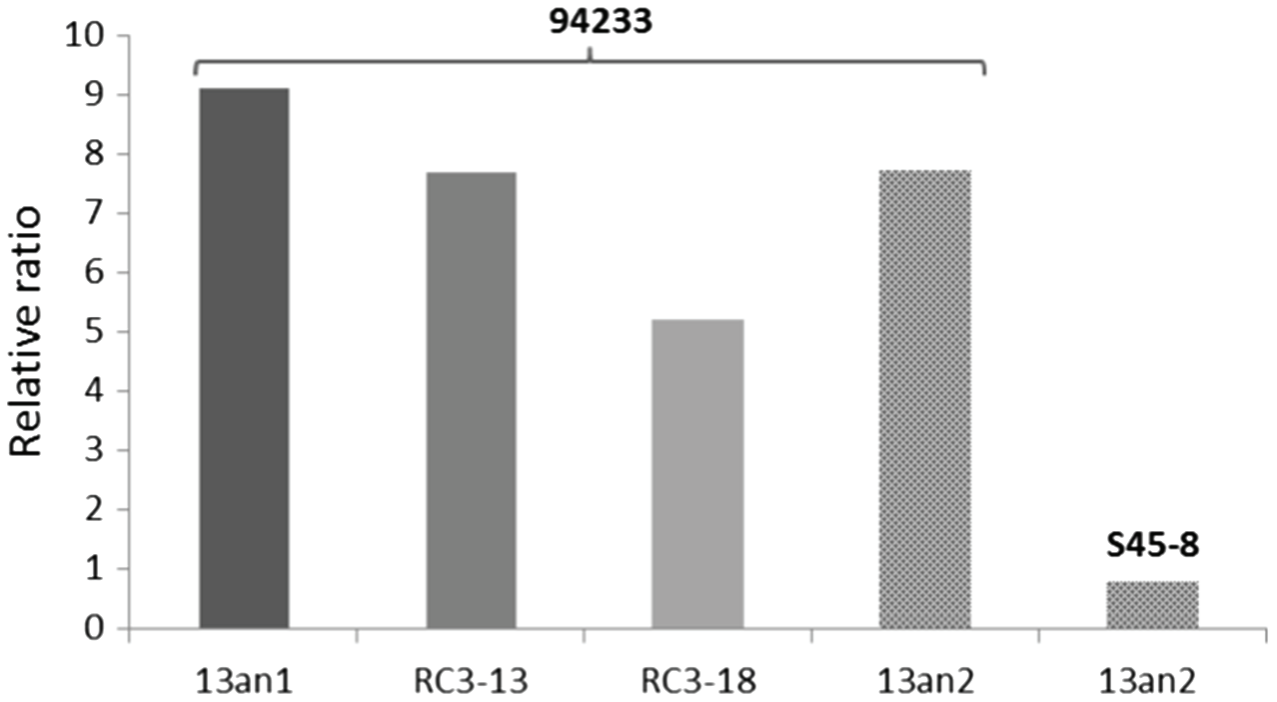
Variation in the ratio of viral RdRp and CP in different host strains. Strains 94233 and 94233-RC3 hosted virus HetPV13-an1, and strains 94233-PV13-an2 and S45-8 hosted virus HetPV13-an2. 13an1=HetPV13-an1 and RC3-13=94233-RC3 before storage; RC3-18=94233-RC3 after storage, 13an2=HetPV13-an2.

The relative ratios of RdRp to CP were somewhat lower (7.7 and 5.2 before and after the long storage, respectively) in 94233-RC3 and 94233-PV13-an2 (7.7) compared to 94233 (8.7). Moreover, isolates 94233-RC1 and 94233-RC2 that recovered their growth rates temporarily had lowered levels of transcripts amounts (Supplementary Figure 1) and also had a significantly higher RdRp to CP ratios of 27 and 17, respectively (Supplementary Figure 2). In contrast, the ratio of RdRp and CP transcripts of HetPV13-an2 was about 90% lower in its original host S45-8 than in 94233 (Figure 5).

### Growth Rates

**Figure 6:**
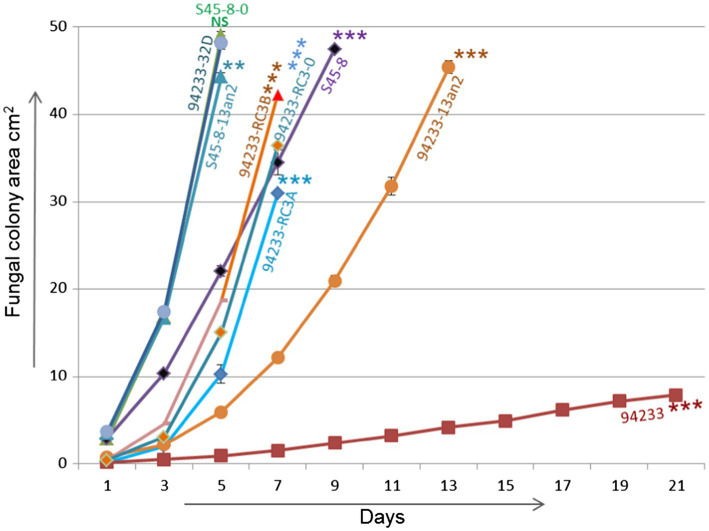
Growth rate analysis. Growth rates of 94233-RC3 and its stored derivative 94233-RC3B infected with HetPV13-an1 including original 94233 with its consistent reduced phenotype and the partitivirus-free control 94233-32D (Kashif et al., 2019). 94233-RC3A=before storage; 94233-RC3B=after storage; 94233 (the original slow-growing strain hosting HetPV13-an1); S45-8-0=virus-free control; S45-8-PV13an2 (single virus infection HetPV13-an2); S45-8 (the original strain with HetPV13-an2 and HetPV7-an1); and 94233-PV13an2 (HetPV13-an2 hosted by 94233 strain). The significance of the differences between the mean growth per day of the virus-free/cured and virus infected fungal isolates is shown by asterisks: ^*^p<0.05; ^**^p<0.001; and ^***^p<0.0001; #=single replicate; NS=Non-significant.

Inoculums made from single 2% MEA plate in order to create 12 independent biological replicates of 94233-RC3 before and after storage 94233-RC3 appeared to be stable (Supplementary Figure 3) and had growth rates that were 45 and 62% of partitivirus-free 94233-32D and 12 and 16 times higher than the original HetPV13-an1 hosting 94233 (Figure 6). The 94233-RC3 strain that had lost HetPV13-an1 during the storage had a very similar growth rate as the virus-hosting strain 94233-RC3 before and after the storage.

94233-PV13-an2 showed a significant 64% growth reduction compared to 94233-32D, but S45-8-an2 had only 9.6% growth reduction compared to the cured S45-8-0 and thus grew at the same rate as 94233-32D. Originally HetPV13-an2 was found in coinfection with HetPV7-an1 in its original host S45-8 and showed similar growth rates as 94233-RC3, i.e., up to 45% slower growth compared to the partitivirus-free S45-8-0 (Figure 6).

## Discussion

This study was launched based on our observation that a *Heterobasidion* strain seriously debilitated due to a HetPV13-an1 infection had recovered spontaneously. That accords with some previous studies on other mycoviruses which have shown that coinfections of two fusarivirus strains of *F. graminearum* are linked with debilitating phenotypic alterations in their fungal hosts and that the effect of *Fusarium* mycoviruses in the fungal transcriptome is virus-specific (Darissa et al., 2012; Lee et al., 2014). In this study, we originally assumed that the fast-growing hyphal sector had been cured of the virus, but after examining the presence of the virus and its genome sequence, it was evident that there was no change in the virus itself. As mutation in the virus genome did not cause the altered phenotype of 94233-RC3, we conducted analyses of gene expression to investigate possible host-related reasons behind the recovery of our original fast-growing sector isolate. In a previously published study, the debilitating infection of HetPV13-an1 seriously affected many different biochemical pathways and the expression of some genes was even turned down completely (Vainio et al., 2018b). Similar analysis of the sector isolate 94233-RC3 revealed that the fungus had restored most of its gene regulation despite the presence of HetPV13-an1, including the two extremely downregulated (practically knocked out) genes in 94233. However, notably high changes were still observed in single genes related to the formation of fungal hypha, monooxygenase activity, and carbohydrate metabolism, although it remained unclear how and why the expression of these three genes was retained in the sector isolate 94233-RC3.

The recovery of most gene functions in 94233-RC3 was accompanied by a drastic drop in the quantities of viral transcripts, i.e., the viral titer: The amounts of RdRp and CP transcripts of HetPV13-an1 in 94233-RC3 were 74 and 68% lower, respectively, than in seriously debilitated 94233. In *F. oxysporum* and *Lentinula edodes*, the viral titer correlates with the degree of debilitation (Kim et al., 2015; Lemus-Minor et al., 2018; Yu and Kim, 2021), but there is also evidence that viral titers depend on host cell physiology (Schoffelen et al., 2013), culture conditions (Hillman et al., 1990), and environmental factors (Bryner and Rigling, 2011). Also RNA silencing could have a role in controlling the virus titers and associated phenotypic changes as, e.g., Mochama et al. (2018) showed that phenotypic alterations caused by virus infection are driven by RNA silencing mechanisms in *Sclerotium sclerotiorum* and antiviral RNA silencing responses led to phenotypic changes of fungal hosts in *Cryphonectria parasitica* (Segers et al., 2007) and *Colletotrichum higginsianum* (Campo et al., 2016). The expression of genes for RNA silencing was only relatively slightly affected by the debilitating HetPV13-an1 infection of 94233 (Vainio et al., 2018b), and the sector isolate 94233-RC3 did not differ much from that. Therefore, our results support the view that RNA silencing is not much affected by an infection of HetPV13-an1.

Jurvansuu et al. (2014) suggested that the ratio of RdRp and CP transcripts of partitiviruses would be more important than their actual quantities in determining the phenotypic effects on the host. In this study, we observed slightly lower ratios of virus-encoded transcripts in 94233-RC3 and also in 94233-PV13-an2 hosting a closely related virus strain HetPV13-an2 causing only mild symptoms. Furthermore, the quantities of the two transcripts were almost equal in the original host S45-8 with a somewhat debilitating coinfection of HetPV13-an2 and HetPV7-an1, and highly similar to HetPV13-an1 in the recovered sector isolate 94233-RC3. These findings may be taken as support for the hypothesis by Jurvansuu et al. (2014), but also the direct transcript quantities correlate with the degree of symptoms. However, it has recently been described that RdRp transcripts in partitiviruses are often more abundant than CP transcripts although these infections seem to lack debilitating effects (Kashif et al., 2019; (Vainio et al., 2015a), which seems to contradict with the hypothesis of Jurvansuu et al. (2014).

94233-RC3 isolates before and after storage (2018) did not show significantly different growth rates. However, one of the replicates from the storage was free of HetPV13-an1, which might have been due to uneven distribution of viruses in the mycelium. The growth rate of this isolate 94233-RC3-0 was similar to 94233-RC3 strains that had retained the virus. This might suggest that the recovered phenotype of 94233-RC3 is due to a genetic or other change in the host mycelium that provides tolerance against the debilitating effects of HetV13-an1 but at the same time reduces the inherent growth rate of the mycelium. Non-uniform occurrence of virus particles within fungal mycelia has been reported by Ghabrial (1980), but in contrast to our observation, virus-like particles were observed more frequently in old than in actively growing hyphal tips by Border et al. (1972) and DeMarini et al. (1977).

A single infection of HetPV13-an2 was cryptic in its native host but caused considerable debilitation in a non-native host. This was in accordance to a previous study (Jurvansuu et al., 2014) where virus strains in exotic hosts seemed to have more significant phenotypic effects than in their native hosts. However, a coinfection with another virus strain HetPV7-an1 (S45-8) slowed down the growth by 45% after inoculation. This accords also with analyses by Kashif et al. (2019), who showed that different strains of HetPV11 affected differently the debilitating effects of HetPV13-an1. Interactions between viral double infections were also studied by Wu et al. (2010), who described suppression of replication of Botrytis cinerea mitovirus 1 (BcMV1) by another related RNA virus (BcMV1-S). Also in *Rosellinia necatrix,* the phenotypic outcomes were different when the fungus was hosting single or coinfections of a megabirnavirus and a partitivirus (Sasaki et al., 2016). Moreover, phenotypic variations caused by interplay between partitiviruses (Telengech et al., 2020) and in a coinfection of a chrysovirus and a partitivirus in the human pathogen *A. fumigatus* caused unique phenotypic modifications (Bhatti et al., 2011).

The recovered HetPV13-an1 infected sector isolate 94233-RC3 was originally isolated and studied for its growth rate as well as for fungal and viral gene expressions almost a decade ago (but reported only here). In this study, we found that the virus infection in the sector isolate was low titer and susceptible/vulnerable to storage and culturing conditions. As HetPV13-an1 and other Heterobasidion partitiviruses have previously been stored in similar conditions for several years and even more than half a century (Kashif et al., 2015), this loss of a virus was surprising and suggests that the balance between the host and virus in 94233-RC3 was somewhat less stable than normally. However, loss of fungal viruses during a storage has previously been observed in other fungi, such as *C. parasitica* and *Fusarium circinatum* (Springer et al., 2013; Zamora-Ballesteros et al., 2021).

## Conclusion

This study showed that *Heterobasidion* strains infected by a single partitivirus may show different phenotypes with considerably different fungal transcriptomes, viral titers, transcript ratios, and mycelial appearances.

## Data Availability Statement

The datasets presented in this study can be found in online repositories. The names of the repository/repositories and accession number(s) can be found in the article/Supplementary Material.

## Author Contributions

MK, EV, and JH conceived and designed the experiments. MK, JJ, and RH performed the experiments. MK, JJ, EV, and JH analyzed the data and wrote the paper. All authors contributed to the article and approved the submitted version.

## Funding

This work was supported by the Academy of Finland (grant numbers 322001 and 309896).

## Conflict of Interest

The authors declare that the research was conducted in the absence of any commercial or financial relationships that could be construed as a potential conflict of interest.

## Publisher’s Note

All claims expressed in this article are solely those of the authors and do not necessarily represent those of their affiliated organizations, or those of the publisher, the editors and the reviewers. Any product that may be evaluated in this article, or claim that may be made by its manufacturer, is not guaranteed or endorsed by the publisher.

## Footnotes

^1^ http://www.macrogen.com

^2^ http://genome.jgi.doe.gov/Hetan2/Hetan2.home.html

^3^ http://rest.gene-quantification.info/
