# Supplementary material for: Phenotypic Recovery of a Heterobasidion Isolate Infected by a Debilitation-Associated Virus Is Related to Altered Host Gene Expression and Reduced Virus Titer
Source: Front Microbiol. 2021 Oct 14;12:661554. doi: 10.3389/fmicb.2021.661554 (PMC8930199; doi:10.3389/fmicb.2021.661554)
Supplement: Supplementary file 1 [file Table_1.docx]

Supplementary Material

# Supplementary Data

Supplementary Material should be uploaded separately on submission. Please include any supplementary data, figures and/or tables. All supplementary files are deposited to FigShare for permanent storage and receive a DOI.

Supplementary material is not typeset so please ensure that all information is clearly presented, the appropriate caption is included in the file and not in the manuscript, and that the style conforms to the rest of the article. To avoid discrepancies between the published article and the supplementary material, please do not add the title, author list, affiliations or correspondence in the supplementary files.

# Supplementary Figures and Tables

For more information on Supplementary Material and for details on the different file types accepted, please see [here](http://home.frontiersin.org/about/author-guidelines#SupplementaryMaterial). Figures, tables, and images will be published under a Creative Commons CC-BY licence and permission must be obtained for use of copyrighted material from other sources (including re-published/adapted/modified/partial figures and images from the internet). It is the responsibility of the authors to acquire the licenses, to follow any citation instructions requested by third-party rights holders, and cover any supplementary charges.

## Supplementary Figures

**
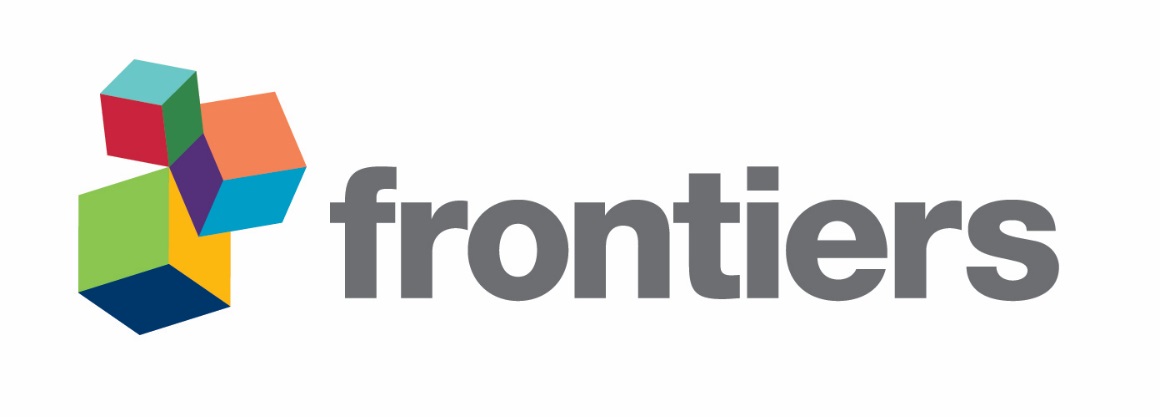
**

**Supplementary Figure 1.** The figure legends are required to have the same font as the main text, 12 point normal Times New Roman, single spaced. Please use a single paragraph for each legend and prepare the figures keeping in mind the PDF layout.

**
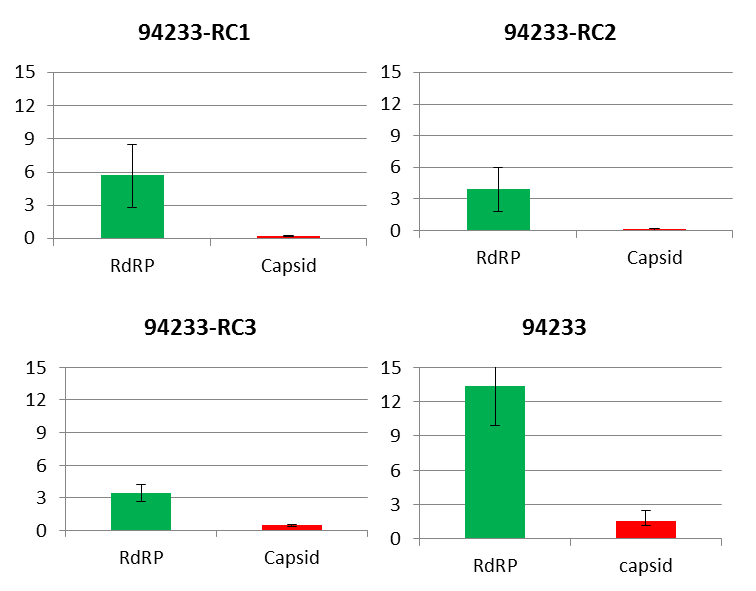
**

**Supplementary Figure 1.** The variation in viral CP and RdRp transcript levels in *H. annosum* strains infected by partitiviruses. Fast growing sector isolates 94233-RC3 with infection by HetPV13-an1 and Strain 94233 with its natural viral infection HetPV13-an1.

**Supplementary Figure 2.** Variation in the ratio of viral RdRp and CP in host strain 94233. 94233-RC (1-3) cured isolates hosted virus HetPV13-an1.

**
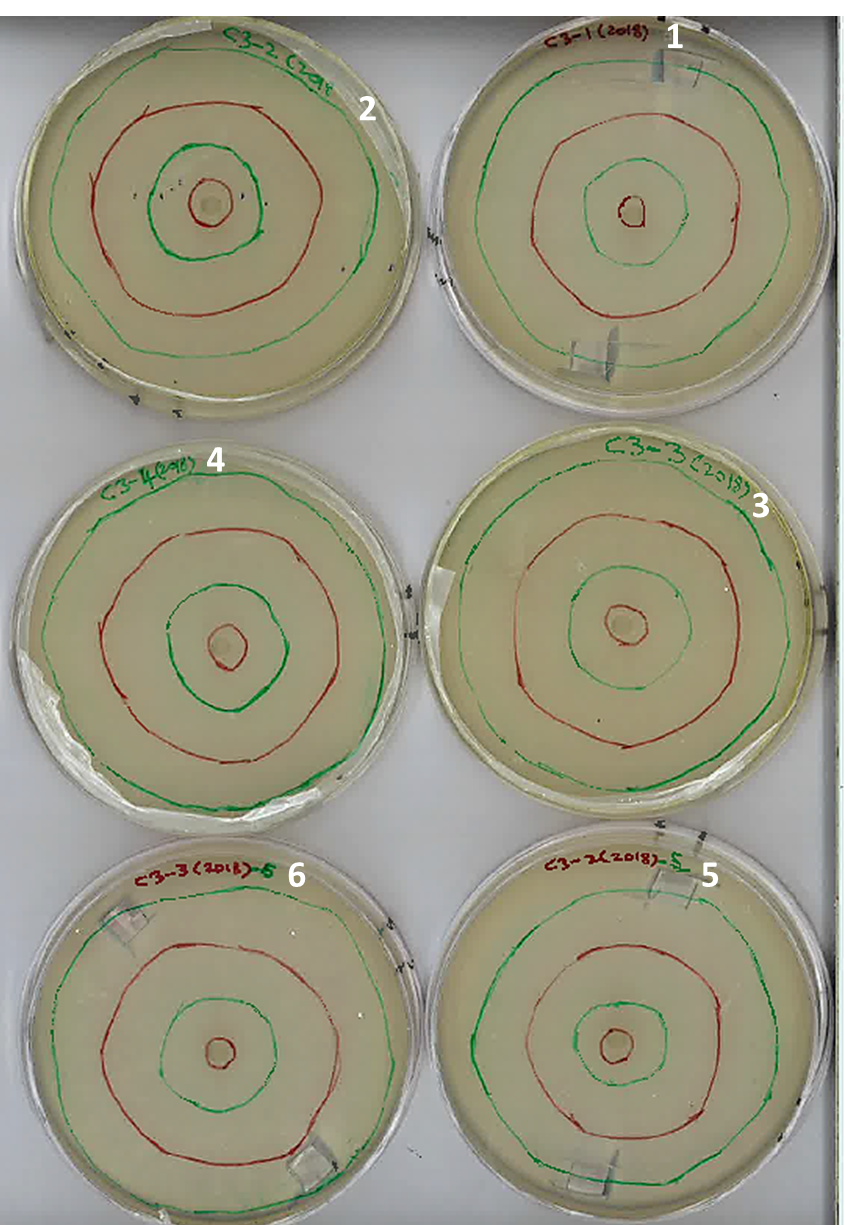
**

**
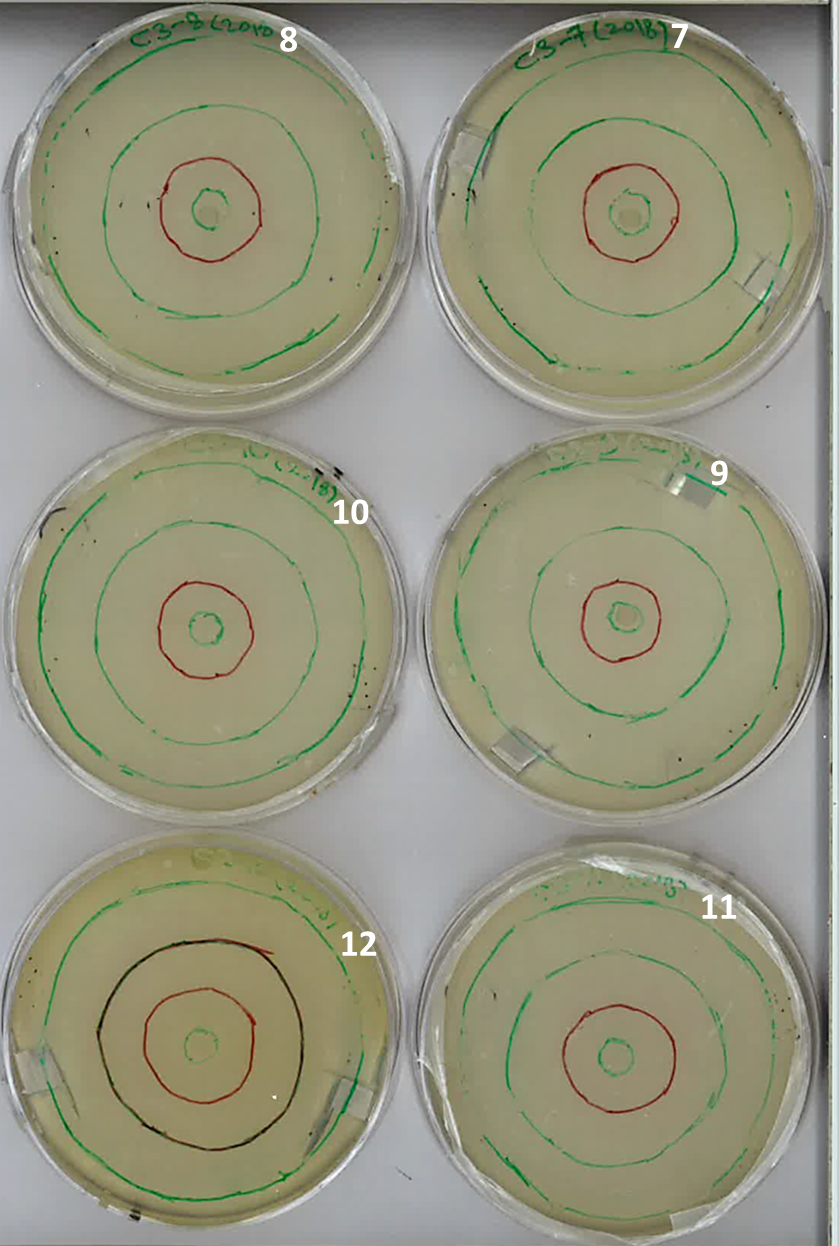
**

**Supplementary Figure 3.** Growth rates for 94233-RC3 after long storage at 4°C containing 12 replicates.

**Supplementary Table S1.** **Mapping statistics for the RNA-Seq data.** Alterned phenotype *H. annosum* C3 (94233) is infected with HetPV13-an1 and 94233/32D is a partitivirus-free isogenic strain, and three biological replicates were used for each strain.

| Sample ID | Number of reads | Number of reads mapped to reference genome | Percentage of mapped reads from all reads | Uniquely aligned reads | Reads mapped to more than one genome location | Reads not mapped to reference genome | Percentag of uniquely mapped reads | Reference |
| --- | --- | --- | --- | --- | --- | --- | --- | --- |
| 94233-RC3_1 | 17833961 | 12501698 | 70.10 % | 12197769 | 303929 | 5332263 | 97.6 % | This study |
| 94233-RC3_2 | 16720416 | 11331524 | 67.77 % | 11069240 | 262284 | 5388892 | 97.7 % |  |
| 94233-RC3_3 | 16114116 | 11005531 | 68.30 % | 10792269 | 213262 | 5108585 | 98.1 % |  |
| 94233/32D_1 | 21666839 | 14917271 | 68.85% | 14570928 | 346343 | 6749568 | 97.7 % | Vainio et al., 2018b |
| 94233/32D _2 | 18132529 | 12587443 | 69.42% | 12301201 | 286242 | 5545086 | 97.7 % |  |
| 94233/32D _3 | 17921554 | 12437311 | 69.40% | 12160000 | 277311 | 5484243 | 97.8 % |  |

**Supplementary Table 2**. List of target genes (TG) selected from RNA-seq date and validation of their FC expression based on RT-qPCR with their mean reaction efficiencies.

|  | RNA-Seq  *H. annosum* |  |  | RNA-seq based gene expression | | | | RT-qPCR validation *H. annosum* | | |
| --- | --- | --- | --- | --- | --- | --- | --- | --- | --- | --- |
|  |  |  |  | 94233 | | **94233-RC3** | |  | 94233 | **94233-RC3** |
| TG | Gene prediction | Gene ID in  *H. annosum* genome v.2.0 at JGI | Functional category | FC^1^ | FDR  *p*-value^2^ | FC^1^ | FDR  *p*-value^2^ | E^3^ | FC^4^ | **FC^5^** |
| 1 | Carbohydrate-binding module family 19 | fgenesh1_pm.02_#_834 | Carbohydrate metabolism | 78.99 | 1.96 e-04 | **10.61** | 1.94 e-02 | 1.71 | 30.74 | **8.12** |
| 2 | RTA1-domain-containing protein | Hetan1.estExt_Genewise1Plus.C_40642 | Detoxification | 13.51 | 2.30 e-05 | **2.69** | 2.69 e-02 | 1.73 | 7.35 | **2.77** |
| 3 | Tetraspanin family protein | gw1.13.1244.1 | Cell wall/ membrane | 13.80 | 4.52 e-04 | **3.74** | 4.97 e-02 | 1.71 | 11.87 | **1.34** |
| 4 | Aryl-alcohol oxidase 9, aao9 | Hetan1.estExt_Genewise1Plus.C_130459 | Redox | 20.14 | 6.23 e-05 | **3.41** | 3.23 e-02 | 1.72 | 16.99 | **3.86** |
| 5 | Cytocrhome P450 | estExt_Genemark.C_120425 | Detoxification | 158.4 | 1.03 e-06 | **-3.10** | 4.43 e-02 | 1.73 | 9.37 | **-2.04** |
| 6 | MFS amino acid permease | Hetan1.fgenesh2_pm.C_scaffold_2000190 | Amino acid metabolism | 13.00 | 5.14 e-06 | **1.705** | 7.39 e-02 | 1.72 | 9.41 | **2.26** |
| 7 | Pentafunctional AroM protein | e_gw1.13.1032.1 | Amino acid metabolism | 8.47 | 9.94 e-04 | **1.87** | 2.09 e-01 | 1.69 | 4.99 | **1.54** |
| 8 | UDP-glucose 4-epimerase | estExt_Genewise1Plus.C_010240 | Carbohydrate metabolism | 14.85 | 1.64 e-05 | **1.24** | 4.26 e-01 | 1.69 | 10.53 | **1.42** |
| 9 | Citrate synthase | Genemark.3471_g | Mitochondrial energy production | 7.85 | 1.92e-05 | **1.60** | 8.24 e-02 | 1.73 | 17.68 | **2.12** |
| 10 | IucA/IucC | estExt_fgenesh1_pg.C_040344 | Inorganics metabolism | 16.53 | 6.53 e-05 | **-1.61** | 3.66 e-01 | 1.72 | 21.90 | **1.29** |
| 11 | Catalase | Hetan1.estExt_Genewise1Plus.C_61257 | Detoxification | 35.32 | 2.81 e-04 | **3.85** | 8.40 e-02 | 1.72 | 22.78 | **4.12** |
| 12 | Fruit-body specific gene C | Hetan1.estExt_fgenesh2_pm.C_70066 | Sex related | 12.90 | 4.27 e-04 | **2.82** | 1.25 e-01 | 1.72 | 10.75 | **1.54** |
| 13 | MFS glucose transporter 1 | Hetan1.estExt_Genewise1Plus.C_100682 | Carbohydrate metabolism | -19.4 | 2.60 e-05 | **-3.84** | 3.73 e-03 | 1.81 | -13.5 | **-2.27** |
| 14 | Lignin expressed protein-lep1 | Hetan1.Genemark.6963_g | Carbohydrate metabolism | -7.02 | 1.79 e-04 | **-2.80** | 1.32 e-02 | 1.75 | -3.08 | **-1.11** |
| 15 | Glycoside Hydrolase Family 3 protein, gh3.3 | Hetan1.Genemark.8846_g | Carbohydrate metabolism | -11.0 | 9.36 e-05 | **-2.50** | 2.04 e-02 | 1.72 | -5.62 | **-2.15** |
| 16 | Serine/threonine kinase | gw1.08.1665.1 | Cell cycle control | **-9.52** | 3.46 e-04 | **-2.84** | 4.31 e-02 | 1.71 | 1.00 | **1.09** |
| 17 | STE20-like serine/threonine kinase | fgenesh1_pm.04_#_184 | Cell cycle control | **-594** | 2.22 e-06 | **-2.64** | 5.63 e-03 | 1.71 | nd | **-1.97** |
| 18 | RNA-directed RNA polymerase QDE-1 | estExt_fgenesh1_pg.C_020714 | RNA related | -12.8 | 8.26 e-05 | **-3.50** | 9.51 e-03 | 1.73 | -8.55 | **-3.40** |
| 19 | Formin homology protein | Hetan1.fgenesh2_pg.C_scaffold_13000145 | Cell cycle control | -11.6 | 4.51 e-05 | **-2.61** | 1.18 e-02 | 1.77 | -18.9 | **-3.12** |
| 20 | Piwi-domain containing protein | Hetan1.EuGene9000385 | RNA related | **-17.9** | 2.12 e-04 | **-2.51** | 4.33 e-02 | 1.82 | **-333** | **-3.97** |
| 21 | HD1 homeodomain transcription factor, mating type protein | Hetan1.EuGene1000636 | Sex related | -1388 | 4.71 e-07 | **-2.68** | 1.28 e-03 | 1.69 | nd | **-2.55** |
| 22 | Transcription factor of the Forkhead/HNF3 family | gw1.04.2473.1 | Transcription | -15.1 | 2.20 e-04 | **-4.01** | 2.27 e-02 | 1.72 | -6.85 | **-1.12** |
| 23 | Glycoside Hydrolase Family 7 protein cellobiohydrolase | Hetan1.estExt_Genewise1.C_110899 | Carbohydrate metabolism | **-613** | 3.10 e-04 | **-2.65** | 2.71 e-01 | 1.71 | -200 | **8.70** |
| 24 | Mitotic spindle checkpoint protein | e_gw1.01.3559.1 | Cell cycle control | -23.8 | 2.49 e-05 | **-2.00** | 5.28 e-02 | 1.67 | -3.00 | **1.44** |
| 25 | Hydrophobin 2 HAH2 | Hetan1.estExt_fgenesh3_kg.C_90130 | Cell wall/ membrane | -53.5 | 3.15 e-05 | **-1.16** | 7.62 e-01 | 1.70 | -20.8 | **1.54** |
| 26 | Cytochrome P450 monooxygenase 8 cyp8 | Hetan1.estExt_Genewise1Plus.C_70955 | Detoxification | **-539** | 3.33 e-05 | **-1.02** | 9.40 e-02 | 1.78 | -5.13 | **1.46** |
| 27 | Piwi-domain containing protein | Hetan1.estExt_fgenesh2_pm.C_90173 | RNA related | -19.2 | 1.23 e-04 | **-1.86** | 9.81 e-02 | 1.71 | -10.9 | **-2.43** |
| 28 | zfp5 (zinc finger domain containing protein | Hetan1.EuGene4000639 | Transcription | -13.3 | 1.63 e-04 | **-1.66** | 1.34 e-01 | 1.67 | -4.95 | **1.12** |
| **Dicer** | | Hetan1.Genemark.5915_g | RNAi related | -1.90 | 9.26 e-05 | **-1.26** | 5.16 e-02 | 1.70 | -1.18 | **-1.7** |
|  |  | e_gw1.03.2068.1 |  | -1.22 | 9.79 e-02 | **-1.18** | 1.52 e-01 | 1.71 | -1.25 | **-5.95** |
| **Argonaute** | | e_gw1.02.2254.1 | RNAi related | 1.88 | 7.41 e-05 | **1.235** | 7.44 e-02 | 1.72 | -1.13 | **-2.12** |
|  |  | Hetan1.estExt_Genewise1.C_80495 |  | -2.44 | 1.39 e-01 | **-1.21** | 7.51 e-01 | 1.69 | 1.065 | **1.48** |
|  |  | estExt_Genewise1.C_033087 |  | -5.95 | 3.57 e-03 | **-1.92** | 1.88 e-01 | 1.70 | 1.249 | **-1.56** |
|  |  | estExt_fgenesh1_pg.C_030285 |  | -1.64 | 3.78 e-02 | **-1.34** | 9.01 e-02 | 1.71 | 2.248 | **-3.31** |

^1^ Expression ratio FC (fold change) between *H. annosum* 94233, recovered 94233-RC3 (shown bold) and 94233/32D. Positive values indicate upregulated genes and negative values downregulated genes.

^2^ False discovery rate adjusted *p*-value for the gene expression fold change between *H. annosum* 94233, 94233-RC3 and 94233/32D.

^3^ Average reaction efficiency (E) of all replicates (*H. annosum*) based on Comparative Quantitation Analysis in RT-qPCR. REST 2009 (Relative Expression Software Tool) was used to estimate up and down regulation of gene expression, where REST 2009 RG mode require "Amplification" (E) and take-off values to conduct randomization algorithm.

^4^ Fold change based on relative expression ratios at take-off points normalized with three reference genes for *H. annosum*. Two highly downregulated target genes (17, 21) did not yield any amplification products from *H. annosum* 94233 and therefore the fold change could not be determined (nd).

^5^ Fold change based on relative expression ratios at take-off points normalized with three reference genes for recovered C3.

Note that above mentioned RNA-seq and validation data related to 94233 (HetPV13-an1) has been previously published by Vainio et al., 2018b.

**Supplementary Table S3.** Statistical analysis by *t*-Test (two sample assuming unequal variances) in Excel 2010 to analyse the significance of variations in growth/day among 12 independent isolates (subcultures) for each sample for the growth rate. P-value; H_0_:µ_1_=µ_2 and_ H_1_:µ_1_≠µ_2._ α = 0.05.

| Virus strains | S45-8-0 | 94233-32D | 94233 | 94233-RC3 (before storage)^3^ | 94233-RC3 (after storage)^3^ | 94233-13an2 | S45-8 | S45-8-13an2 |
| --- | --- | --- | --- | --- | --- | --- | --- | --- |
| Growth/day (cm^2^)^1^ | 9.6467 | 9.8096 | 0.3757 | 4.4247 |  | 3.4925 | 5.2771 | 8.8645 |
| S45-8-0 |  | **0.3881 (NS)** |  | <0.0001 | <0.0001 | <0.0001 | <0.0001 | <0.0001 |
| 94233-32D | NS |  | <0.0001 | <0.0001 | <0.0001 | <0.0001 | <0.0001 | <0.0001 |
| 94233 | <0.0001 | <0.0001 |  | <0.0001 | <0.0001 | <0.0001 | <0.0001 | <0.0001 |
|  |  |  |  |  |  | <0.0001 |  | **<0.001** |
|  |  |  |  |  |  |  |  |  |
| 94233-RC3-before storage | <0.0001 | <0.0001 | <0.0001 |  | NS | <0.001 | **0.3525 (NS)** | <0.0001 |
| 94233-RC3 (after storage) |  |  |  | **NS** |  | <0.0001 | NS |  |
| 94233-13an2 | <0.0001 | <0.0001 | <0.0001 | <0.0001 | <0.0001 |  | <0.0001 | <0.0001 |
| S45-8 | <0.0001 | <0.0001 | <0.0001 | <0.0001 | <0.0001 | <0.0001 |  | <0.0001 |
| S45-8-13an2 | <0.001 | <0.0001 | <0.0001 | <0.0001 | <0.0001 | <0.0001 | <0.0001 |  |

^1^ Measurements of growth per day (cm^2^) related to 94233, 94233-RC3 (before/after storage) and HetPV13-an2 in S45-8 and 94233.

**Supplementary Table S4. List of RNA-Seq fold changes (FC), reference gene names and probable functions for the upregulated (positive FC) and downregulated (negative FC) transcripts related to differentially expressed genes detected in this study.** The gene names refer to the *H. annosum* v.2.0 genome sequence deposited at the JGI (http://genome.jgi.doe.gov/Hetan2/Hetan2.home.html).

| No. | FC | Name | Protein | Function |
| --- | --- | --- | --- | --- |
| 1 | 587.68^1^ | estExt_Genewise1Plus.C_080637 | Myosin class II heavy chain | Fungal hyphal growth, septation and conidial germination |
| 2 | 387.23 | Hetan1.EuGene10000523 | Cytochrome P450 CYP2 subfamily | monooxygenase activity |
| 3 | 180.05 | e_gw1.12.503.1 | Sorbitol dehydrogenase | Secondary metabolites biosynthesis, transport and catabolism |
| 4 | 70.586 | fgenesh1_kg.07__94__0_0_A95E1_CCOZ_CCPA_CCPB_CCPC_EXTA | - | - |
| 5 | 32.805 | fgenesh1_pm.07__10 | - | - |
| Down-regulated genes | | |  |  |
| 1 | -10.82 | fgenesh1_kg.03__767__4245_1_CCOZ_CCPA_CCPB_CCPC_EXTA | - | - |
| 2 | -4.926 | Hetan1.e_gw1.5.283.1 | putative L-fuculose-phosphate aldolase (fucA) | Carbohydrate metabolism,  Fructose and mannose metabolism |
| 3 | -4.558 | Hetan1.estExt_fgenesh2_pg.C_100320 | phenol 2-monooxygenase | Detoxification,  Aromatic compound metabolism |
| RNAi-related genes | | |  |  |
| 1 | -1.26 | Hetan1.Genemark.5915_g | dsRNA-specific nuclease Dicer and related ribonucleases | nucleic acid binding, RNA processing |
| 2 | -1.178 | e_gw1.03.2068.1 | dsRNA-specific nuclease Dicer and related ribonucleases | RNA binding, RNA processing |
| 3 | 1.235 | e_gw1.02.2254.1 | Translation initiation factor 2C (eIF-2C) and related proteins, Argonaute | nucleic acid binding, proteolysis |
| 4 | -1.206 | Hetan1.estExt_Genewise1.C_80495 | Translation initiation factor 2C (eIF-2C) and related proteins, Argonaute | nucleic acid binding, proteolysis |
| 5 | -1.92 | estExt_Genewise1.C_033087 | Translation initiation factor 2C (eIF-2C) and related proteins, Argonaute | nucleic acid binding, proteolysis |
| 6 | -1.34 | estExt_fgenesh1_pg.C_030285 | Translation initiation factor 2C (eIF-2C) and related proteins, Argonaute | nucleic acid binding, proteolysis |

**^1^** Expression data in green shows significant FC expression data over 4 with probable gene functions.

**^2^** FC expression data in red corresponds to FC expression data with unknown gene functions.
